# 1015-A. Impact of Patient Occupation on Burn Severity and Recovery

**DOI:** 10.1093/jbcr/irag033.204

**Published:** 2026-04-06

**Authors:** Mahi Pachgade, Christopher J Fedor, Sarah M Tepe, Mare G Kaulakis, Mia Carrarini, Jenny A Ziembicki, Francesco M Egro

**Affiliations:** Drexel University College of Medicine, Plainsboro Township, NJ; University of Pittsburgh Medical Center, Department of Plastic Surgery, Pittsburgh, PA; University of Pittsburgh Medical Center, Mercy Burn Center, Pittsburgh, PA; University of Pittsburgh Medical Center, Mercy Burn Center, Pittsburgh, PA; University of Pittsburgh Medical Center, Department of Plastic Surgery, Pittsburgh, PA; University of Pittsburgh Medical Center, Pittsburgh, PA; University of Pittsburgh Medical Center, Pittsburgh, PA

## Abstract

**Introduction:**

Occupational burns often involve greater complexity in management and recovery compared to non-work related injuries, potentially due to injury mechanisms and exposure severity. This study aims to evaluate the impact of occupation and work-related burns on recovery outcomes using data from a national registry spanning a 10-year period.

**Methods:**

A retrospective analysis was conducted using the American Burn Association (ABA) Burn Care Quality Platform (BCQP) registry (2013-2022). Collected data included demographics, burn characteristics, occupation, work-related injury status, progression, and complications. Analysis included chi-squared tests, Kruskal-Wallis tests, and Generalized Linear Models (GLMs).

**Results:**

The study included 74 522 patients across 28 occupational categories, including 32 351 who were unemployed. The mean age of the cohort was 20.5 ± 8.7 years. Most patients identified as White (57.8%; n = 43 044), followed by Black (19.0%; n = 14 143), and 15.8% (n = 11 759) reported as Hispanic or Latino. Compared to unemployed patients, office and administrative workers experienced a 29.2% longer hospital length of stay (LOS; *p*=.04), followed by transportation workers with a 13.2% increase in LOS (*p*=.004). Food service workers and firefighters had significantly shorter LOS, with reductions of 42.0% (*p*<.001) and 41.3% (*p*<.001), respectively. Transportation jobs were associated with a 37.8% higher total body surface area (TBSA) burned (*p*<.001), while food service and firefighting occupations were associated with a 37.8% (*p*=.047) and 31.0% (*p*<.001) lower TBSA, respectively.

**Conclusions:**

Occupation plays an important role in burn recovery outcomes and complications. This study highlights that burn patients employed in physically demanding or high-risk occupations experience longer hospital stays and sustain larger injuries. Findings suggest that the likelihood of occupational exposure and the physical demands of a patient’s occupation may influence injury severity as well as recovery trajectory.

**Applicability of Research:**

Findings from this study may guide future research and clinical decision-making by supporting the development of targeted interventions tailored to a patient’s occupation and associated risk profile.

